# Assessing multiple factors affecting the gut microbiome structure of very preterm infants

**DOI:** 10.1590/1414-431X2023e13186

**Published:** 2023-12-11

**Authors:** Wenlong Xiu, Jiajia Lin, Yanhua Hu, Heng Tang, Shuangchan Wu, Changyi Yang

**Affiliations:** 1 Department of Neonatology, Fujian Maternity and Child Health Hospital College of Clinical Medicine for Obstetrics & Gynecology and Pediatrics, Fujian Medical University, Fuzhou, Fujian Province, China; 2 Key Laboratory of Bioorganic Synthesis of Zhejiang Province, College of Biotechnology and Bioengineering, Zhejiang University of Technology, Hangzhou, China; 3 Institute of Medical Research, Northwestern Polytechnical University, Xian, Shanxi Province, China

**Keywords:** Gut microbiota, Delivery mode, Antibiotic use in pregnancy, Feeding method, 16S rRNA sequencing analysis

## Abstract

The composition and diversity of the gut microbiota are essential for the health and development of the immune system of infants. However, there is limited information on factors that influence the gut microbiota of very preterm infants. In this study, we analyzed factors that affect the gut microbiota of very preterm infants. The stool samples from 64 very preterm infants with a gestational age less than 32 weeks were collected for 16S rRNA gene sequencing. The infants were divided according to the delivery mode, antibiotic use during pregnancy, and feeding methods. The abundance of *Proteobacteria* was high in both cesarean (92.7%) and spontaneous (55.5%) delivery groups and then shifted to *Firmicutes* after the first week of birth. In addition, *Proteobacteria* was also the dominant phylum of infant gut microbiome for mothers with antibiotic use, with more than 50% after the first week of birth. In comparison, the dominant phylum for mothers without antibiotic use was *Firmicutes*. *Proteobacteria* level was also high in breastfeeding and mixed-feeding groups, consisting of more than 90% of the community. By contrast, *Proteobacteria* was the dominant phylum at the first week of birth but then shifted to *Firmicutes* for the formula-fed group. The alterations of gut microbiota in infants can affect their health condition during growth. This study confirmed that the different feeding types, delivery modes, and use of antibiotics during pregnancy can significantly affect the composition of the gut microbiota of very preterm infants.

## Introduction

Gut microbes are closely related to human health. The human gut has a complex, diverse, and dynamic microbial community. The microorganisms that colonize the intestine can directly affect the health conditions of the host through metabolites and secretion of bioactive factors (1). Gut microbiota plays an indispensable role in defending against colonization by intestinal pathogens, promoting host immune system maturation, and regulating host metabolism (2). The establishment of the intestinal flora homeostasis early in life can impact human health (3). During the neonatal period, the formation of the gut microbiome can protect infants from enteric infections, which is critical for building the immune system (3). Previous studies showed that normal intestinal flora including *Bifidobacteria*, *Streptococci*, *Staphylococci*, and *Weyongococci* can promote the absorption of nutrients and avoid lactic acid accumulation in newborns, highlighting the importance of the establishment of the intestinal microecology in infants (4). Studies have shown how altered intestinal microbiota affect infant health and future health outcomes (5,6). However, limited data were found on the factors that influence the gut microbiota structure of very preterm infants.

Currently, the global preterm birth rate has risen to approximately 10%, including 2.3 million extreme preterm infants (those born at gestational age <32 weeks) born each year (7). Due to immature organ development and low immunity, extreme preterm infants often suffer from a variety of complications, such as necrotizing small bowel colitis (NEC), late-onset sepsis (LOS), chronic bronchopulmonary dysplasia (BPD), and intraventricular hemorrhage (IVH) (8). The intestinal flora of preterm infants is established from birth and is susceptible to a variety of perinatal factors, including delivery mode, feeding methods, medication use, living conditions, and many other factors (9).

Most of the early intestinal flora of naturally delivered newborns are originated from the maternal intestine, vagina, skin, and mouth. However, infants born by cesarean delivery do not have the chance to obtain this flora due to the lack of direct contact with their mothers at birth (10). Therefore, the breakdown of gut microbiota can be different in infants with different delivery modes. Studies show that about 25% of women receive a course of antibiotics during pregnancy. In addition, antibiotics account for about 80% of medications prescribed during pregnancy (11), and maternal prenatal antibiotic use is a factor that affects the establishment of neonatal intestinal flora (12). Different feeding methods also have a significant effect on the gut microbial structure of infants and children (13). Breast milk is considered to be the ideal nutrient for the normal growth of infants and children because it is rich in bioactive substances and beneficial microorganisms. Breast milk is suggested to help establish the intestinal flora and the development of the immune system of infants and children (14). Notably, breastfed infants have a significantly lower mortality rate than formula-fed infants (15). However, due to various factors, more and more infants are not breastfed (16).

In this study, we sequenced and analyzed the intestinal flora of very preterm infants using 16s rRNA amplicon sequencing. We systematically investigated the effects of delivery mode, maternal prenatal antibiotic use, and feeding methods on the composition, abundance, and diversity of the intestinal flora of very preterm infants, which can help optimize the care of very preterm infants.

## Material and Methods

### Participants

This study was carried out according to the Declaration of Helsinki with regard to ethical principles for research involving human subjects. The study protocol was approved by the Ethics Committee of Fujian Maternity and Child Health Hospital (2021KLRD09028). The stool samples were collected from very preterm infants (gestational age less than 32 weeks) born in Fujian Maternity and Child Health Hospital from May 2021 to June 2022. The hospitalization data including delivery mode, maternal prenatal antibiotic use, and feeding methods were collected.

The participants were divided into 7 groups according to the influencing factors, with group CD representing cesarean delivery, group SD representing spontaneous delivery, group AP representing antibiotic use during pregnancy, group NAP representing non-antibiotic use during pregnancy, group BF representing breastfeeding, group FF representing formula-fed, and group MF representing mixed-feeding. Informed consent was obtained from the parents or legal guardians of the infants.

### Inclusion and exclusion criteria

Inclusion criteria were gestational age of less than 32 weeks and transferring to neonatology for inpatient treatment immediately after birth. Women with severe asphyxia during delivery, severe intrauterine infections, congenital malformations, and congenital genetic metabolic disorders were excluded. Other exclusion criteria were feeding intolerance, gastrointestinal perforation, necrotizing small intestine colitis, and gastrointestinal malformation.

### Stool sample collection

Stool specimens were collected from preterm infants in the 1st, 2nd, and 3rd weeks of life using disposable sterile stool collection bottles and transferred to -80°C for storage within 2 h.

### Bacterial DNA extraction and sequencing

The stool samples were stored at -40°C freezer for transportation. DNA extraction from stool samples and 16S rRNA gene amplicon sequencing (Illumina-Miseq) were performed by NovoHoZhiYuan (China). To investigate the composition of the gut microbiota, only the variable region (V4) of the 16S rRNA gene was sequenced.

### Bioinformatics analysis

The sequencing data were spliced by FLASH software (Johns Hopkins University, http://ccb.jhu.edu/software/FLASH/), and the chimeras were filtered by Vsearch software (University of Oslo, Norway). Representative sequences with sequence similarity greater than 97% were selected as the same operational taxonomic unit (OTU). Taxonomic analysis was performed based on the RDPclassifier Bayesian algorithm (Michigan State University, https://github.com/rdpstaff/classifier), and based on the results, the diversity index and species composition of each group of intestinal flora were analyzed. BLAST was applied to compare the sequences, and finally species diversity (Simpson's and Shannon's indices) was analyzed using QIIME software (Northern Arizona University, https://qiime2.org/) using principal component analysis (PCA). Species were analyzed also at the phylum and genus taxonomic levels.

## Results

### Participant characteristics

To control for variables, data were independently collected to research the three key factors including delivery mode, prenatal antibiotic use, and feeding method. In each group, only one of the factors was different. Eighteen had data on delivery mode, of which 9 were delivered spontaneously and 9 had cesarean delivery, all were breastfed after birth, and none of the mothers used antibiotics before delivery. Sixteen infants had data on prenatal antibiotic use, of which 8 with maternal prenatal antibiotic use and 8 without, all were breastfed after birth, and delivered spontaneously. Thirty infants had data on feeding methods, of which 10 were breastfed, 10 were formula-fed, and 10 were mixed-fed, all were delivered spontaneously, and without maternal prenatal antibiotic use. The data are shown in Table 1. There were no statistically significant differences in the mean body mass, mean gestational age, and male-to-female ratio between the 7 study groups.

**Table 1:** Number of infants included in the study of each of the variables and their detailed information.

Variable	Delivery mode(n=18)	Prenatal antibiotic use(n=16)	Feeding method(n=30)
Natural delivery	9	16	30
Cesarean delivery	9	0	0
Maternal prenatal antibiotic use	18	8	0
Non-maternal prenatal antibiotic use	0	8	30
Breastfeeding	18	16	10
Formula-fed	0	0	10
Mixed-feeding	0	0	10
Gender M/F	10/8	9/7	16/14
Gestational age at birth (weeks), mean (SD)	30.42±1.17	29.25±2.32	29.53±1.56
Birth weight (kg), mean (SD)	2.15±0.63	2.42±0.58	2.25±0.67
Age at stool sampling (days), mean (SD)	5.26±1.73	5.35±1.952	5.27±1.452

### Effect of delivery mode on gut microbiota composition

**Figure 1:**
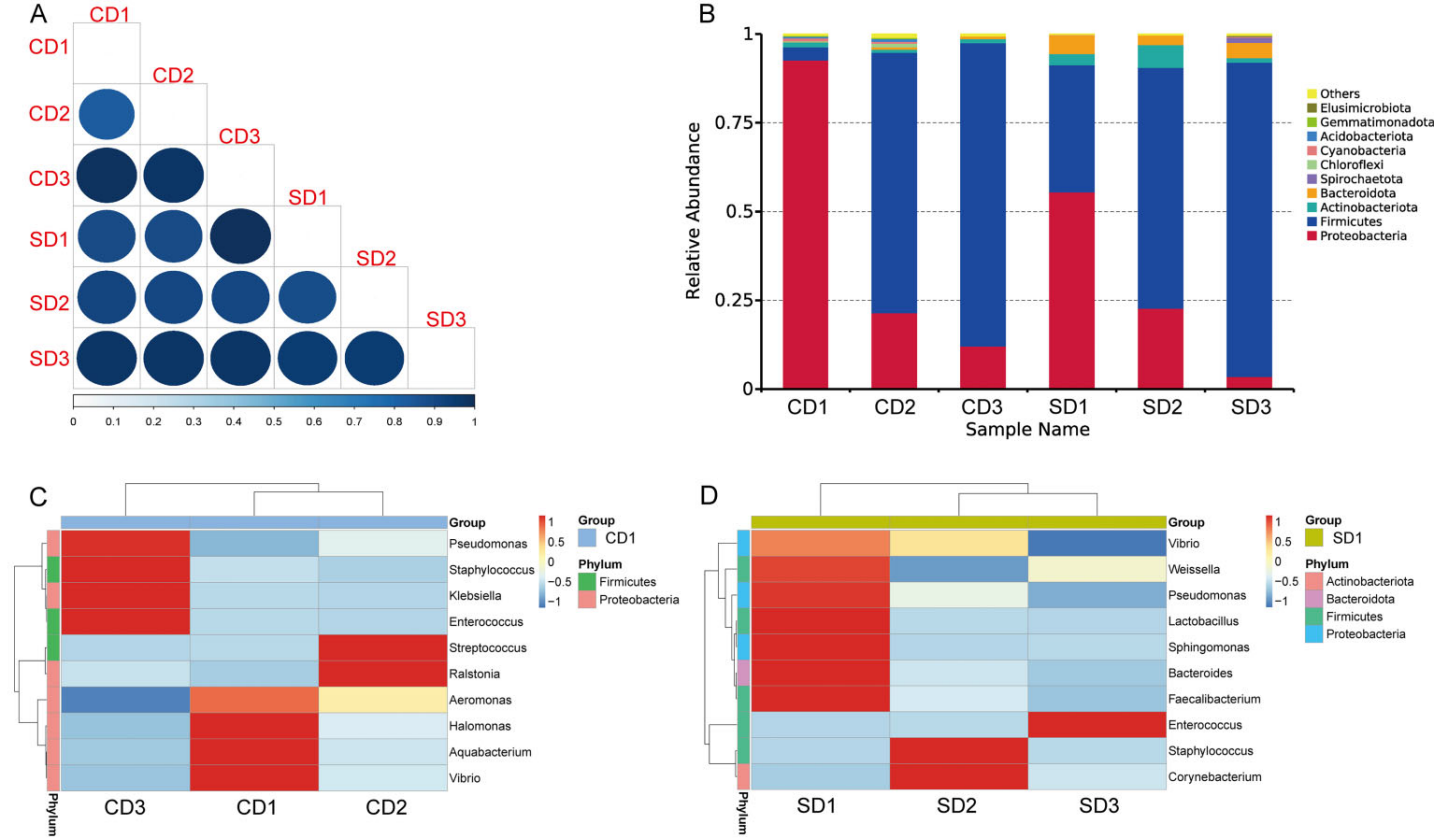
The composition of gut microbiota was affected by delivery mode. **A**, Beta-diversity index of fecal samples at different weeks of age in preterm infants. **B**, Histograms of the distribution of bacterial flora at the portal level in fecal specimens of preterm infants born by caesarian section (CD1, CD2, CD3) and spontaneous delivery (SD1, SD2, SD3) at the 1st, 2nd, and 3rd weeks of life. Microbial community structure at the first, second, and third weeks of cesarean delivery group (**C**) and spontaneous delivery group (**D**).

The distance between species communities is often used to assess the variations of the species between samples (17). In this study, we adopted the beta diversity for the quantification of different communities. We used the Bray-Crutis distance (R language) to assess the community diversity of the cesarean delivery group (group CD) and the spontaneous delivery group (group SD). The value for Bray-Crutis distance is between 0 and 1.0, and the value 0 indicates that there is no difference between the microbial communities of the two samples and the value close to 1 suggests a great difference between the two samples. As seen in Figure 1A, the microbial community structure in the first (CD1), second (CD2), and third weeks (CD3) of group CD were significantly different from those of group SD (as indicated by SD1, SD2, and SD3). Notably, the third week displayed the most significant differences.

The analysis of the microbial community structure of each delivery mode group revealed that 10 phylum existed in stool samples of both group SD and group CD. As shown in Figure 1B, *Proteobacteria* and *Firmicutes* accounted for the largest proportion, especially the *Proteobacteria* in the first week of life (CD1) of group CD, which reached 92.71% and was much higher than that of group SD (SD1=55.53%). The abundance of *Proteobacteria* showed a decreasing trend, the value dropping from 92.71 to 12.10% in group CD. The abundance of *Firmicutes* was relatively low in the first week of life in group CD and group SD, accounting for 3.65 and 35.83%, respectively. However, the abundance of *Firmicutes* gradually became dominant after the first week of life, which reached more than 80% in the third week of life (Figure 1B). Notably, the other phyla accounted for less than 10% of the community in the first three weeks of life, and *Bacteroides* was only found in the three periods of group SD.

Analysis at the genus level revealed that the dominant genera varied across time points in group CD (Figure 1C). The dominant genus in group CD were *Vibrio*, *Aquabacterium*, *Halomonas*, and *Aeromonas* in the first week of life, changing to *Ralstonia* and *Streptococcus* in the second week, and then to *Enterococcus* in the third week (Figure 1C). The dominant genera in group SD was significantly different from group CD. The dominant genus in group SD in the first week were diverse, including *Faecalibacterium*, *Bacteroides*, *Sphingomonas*, *Lactobacillus*, *Pseudomonas*, and *Weissella*, then in the second week became *Corynebacterium* and *Staphylococcus*, and in the third week *Enterococcus* was dominant (Figure 1D).

### Effect of prenatal antibiotic use on gut microbiota composition

**Figure 2:**
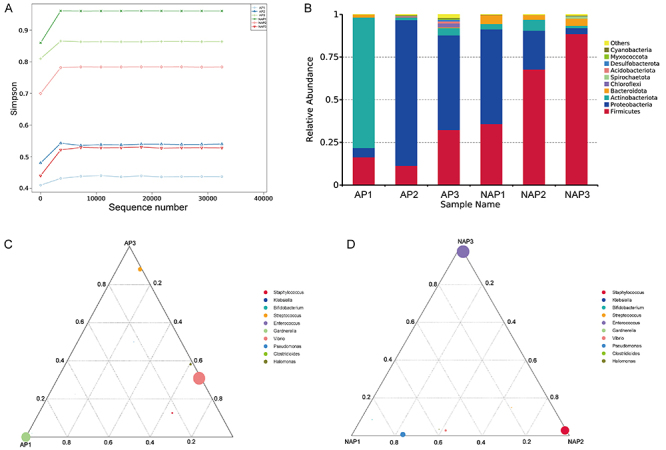
The composition of gut microbiota was affected by maternal prenatal antibiotic use. **A**, Dilution curves (Simpson’s index) of stool samples from preterm infants at different weeks of age (1, 2, and 3) of the maternal prenatal antibiotic use group (group AP) and no maternal prenatal antibiotic uses (group NAP). **B**, Histogram of the bacterial flora abundance at the portal level in fecal specimens of preterm infants in the two groups. Ternary distribution of bacterial flora at the genus level for group AP (**C**) and for group NAP (**D**).

The dilution curve was used to compare the species richness in samples with different amounts of sequencing data (17), in order to indicate whether the amount of sequencing data in the samples was reasonable. As seen in Figure 2A, the curves tended to be stable while the sequence numbers were above 5000, indicating the amount of sequencing data was reasonable, and more data would only produce a small number of new OTUs.

As shown in Figure 2B, 10 phyla were detected in stool samples from very preterm infants whose mothers used antibiotics prenatally (group AP) and not used antibiotics prenatally (group NAP). *Actinobacteriota* was the most abundant phylum in group K at the first week (AP1), which dropped quickly in the second and third weeks. In comparison, *Actinobacteriota* remained low in group NAP. The abundance of *Proteobacteria* decreased over time in group NAP, replaced by the increased abundance of *Firmicutes*. The abundance of *Firmicutes* reached 87.85% at the third week of life in group NAP. In comparison, *Proteobacteria* retained a high level of more than 50% in group AP during the second and third weeks. Except for *Proteobacteria* and *Firmicutes*, the other phyla accounted for less than 15% of the community.

The top 10 species in mean abundance at the genus level were selected to generate a ternary phase diagram. As shown in Figure 2C, the distribution of the points was not concentrated, indicating that the distribution of each genus at different weeks of age was uneven and each stage had its own dominant genus. *Vibrio*, *Gardnerella*, *Streptococcus*, and *Staphylococcus* were the predominant genus in group AP (Figure 2C). Meanwhile, *Gardnerella* was mainly present in the first week, *Vibrio* in the second week, and *Streptococcus* in the third week. *Enterococcus*, *Staphylococcus*, *Pseudomonas*, and *Streptococcus* were the predominant genus in group NAP (Figure 2D). Notably, *Streptococcus* was also the most abundant genus in group AP. Additionally, *Bifidobacterium* was only present in group NAP.

### Effect of feeding methods on gut microbiota composition

**Figure 3:**
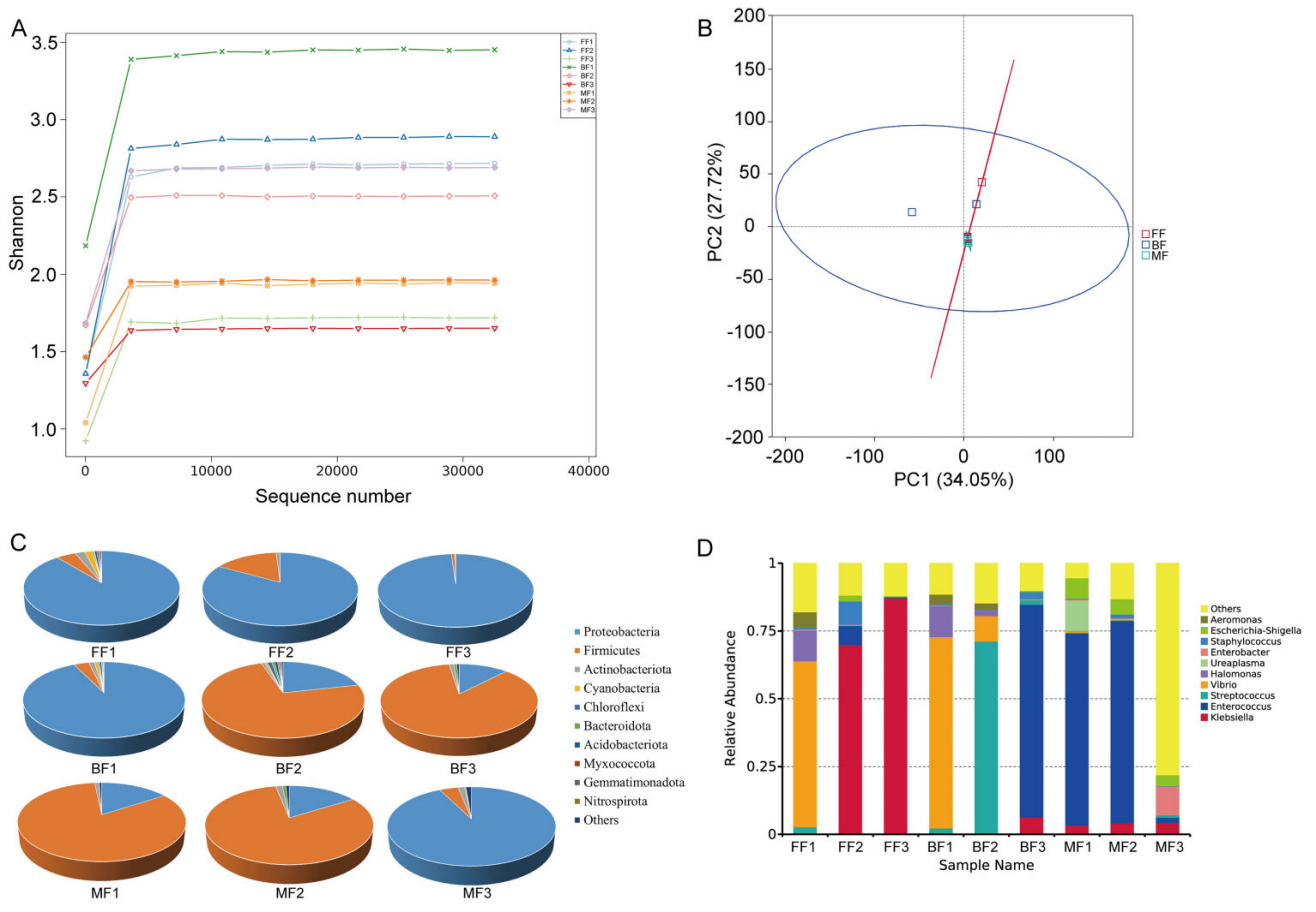
The composition of gut microbiota was affected by feeding methods. **A**, Dilution curves (Shannon index) of intestinal flora sequencing results for the 3 groups of preterm infants (BF: breastfeeding group; FF: formula-feeding group; MF: mixed-feeding group at weeks 1, 2, and 3). **B**, Principal component analysis of gut microbial community of the 3 groups. **C**, Pie charts of intestinal flora at the phylum level of the 3 groups. **D**, Histogram of intestinal flora genera levels in the 3 groups.

The dilution curve showed that all samples displayed a stabilized trend after sequence numbers reached 5000, indicating the sequencing depth met the requirements and the sequenced samples could be used to represent the sample diversity (Figure 3A). The differences and similarities in the structure of the gut microbiota of infants under different feeding methods were characterized using PCA analysis. PCA analysis can help reduce the complexity of the data and facilitate visualization. As shown in Figure 3B, the interpretability of PC1 and PC2 horizontal axes were 34.05 and 27.72%, respectively. The 2 dimensions could explain 61.77% of the differences between the 3 groups (BF, FF, and MF) in our data. The figure also shows that the differences between groups BF and FF are large, but the gut microbiome structure in the MF group remained stable over time.

The gut microbiome structure of preterm infants was analyzed at the phylum level. As shown in Figure 3C, *Proteobacteria* was always the dominant phylum in group BF, and its relative level reached 98.93% of the intestinal flora in the third week. In group FF, *Proteobacteria* accounted for the largest portion in the first week (92.78%), which shifted to *Firmicutes* in the second and third weeks (from 3.63% in the first week to 85.43% in the third week). In group MF, the level of *Firmicutes* was higher in the first and second weeks, accounting for 82.75 and 80.78%, respectively. *Proteobacteria* became dominant in the third week, which reached 92.60%. Comparative analyses of the relative abundance of *Firmicutes* in the three groups revealed that they were mainly present in group BF and group MF. Notably, the other phyla accounted for less than 15% of the community except for *Proteobacteria* and *Firmicutes*.

The gut microbial community of preterm infants was analyzed at the genus level. As shown in Figure 3D, the dominant genera in group BF in the first week was *Vibrio*, accounting for 61.16%, which was similar to group FF. The dominant genera in group FF changed to *Streptococcus* in the second week, but in the third week, the dominant genus was *Enterococcus*. In group MF, *Enterococcus* was the dominant genus in the first two weeks.

## Discussion

In this study, our results showed that delivery mode, maternal prenatal antibiotic use, and feeding method can alter the gut microbiome structure of very preterm infants. The microbial structure of the preterm infant differs significantly from that of term infants and is associated with several serious diseases (18). The dominant phylum in both cesarean and spontaneous delivery groups were *Proteobacteria*, which then changed to *Firmicutes* after the first week of birth, while the ratio of *Proteobacteria*/*Firmicutes* for cesarean delivery group was higher than that of spontaneous delivery. These observations are in agreement with a previous report (19). Several studies declared that NEC and infantile allergic diseases are associated with changes in intestinal flora (20,21). Notably, *Bacteroides* was present in the three periods of the spontaneous delivery group, but it was absent in the cesarean delivery group. *Bacteroides* is important for human health and participate in glycolysis, immune development of the intestinal mucosa, and vitamin synthesis (22). In addition, previous studies show that the reduced relative abundance of *Bacteroides* is associated with childhood obesity (23,24), while cesarean delivery is suggested to increase obesity in children by 30% (25). Furthermore, *Lactobacillus*, which is a probiotic that can regulate human intestinal conditions, only existed in the spontaneous delivery group (26).

The predominant genera in the cesarean group in the third week of birth were all potentially pathogenic, including *Enterococcus*, *Klebsiella*, *Staphylococcus*, and *Pseudomonas*, probably due to the reduced exposure of cesarean-born infants to the normal flora of the mother's birth canal (27). The lower level of beneficial bacteria such as *Bacteroides* and *Lactobacillus* and a lower level of pathogenic bacteria in the intestinal flora of very preterm infants leads to an increased incidence of complications including NEC, LOS, and BPD (28). Naturally delivered babies are exposed to microorganisms in the maternal birth canal and gut, which helps create an environment conducive to the growth of exclusively beneficial anaerobic bacteria at an early age (29).

Antibiotics are drugs that act against bacterial infections (29). Currently, there are growing concerns about the negative effects of antibiotic use, including side effects to the intestinal flora (30). Our results showed that *Actinobacteriota*, *Proteobacteria*, and *Firmicutes* were the dominant phyla, while *Actinobacteriota* accounted for the largest portion of the intestinal flora in the group of mothers that used antibiotics. *Proteobacteria* was the dominant phylum in the three time points in mothers with antibiotic use. A previous study showed that the increased abundance of *Proteobacteria* can lead to increased incidence of NEC (31). Meanwhile, the increase of *Proteobacteria* in the intestine of those infants indicated the unstable structure of the intestinal microbial community or ecological dysbiosis (32). The side effects of prenatal antibiotic use by the mother highlight the need to weigh the pros and cons of prenatal antibiotic therapy.

*Firmicutes* and *Bacteroides* were present in the group of mothers without antibiotic use during pregnancy. *Bacteroides* are a beneficial phylum in the intestine, and *Firmicutes* can decrease the incidence of NEC (33). The dominant genera in the group of mothers with antibiotic use were all potentially pathogenic, including *Vibrio*, *Gardnerella*, *Streptococcus*, and *Staphylococcus*. *Bifidobacterium* is beneficial but was only found in the group of mothers without antibiotic use, suggesting that prenatal use of antibiotics can affect the normal colonization of the intestinal flora of preterm infants. Our results agree with those of a previous study that suggested that prenatal antibiotic exposure delayed *Bifidobacterium* colonization and reduced its relative abundance (34).

The nutritional composition for breast milk and artificial feeding is different (35), which inspired our research to investigate whether feeding types affected the intestinal flora of preterm infants. *Firmicutes* existed mostly in the formula-fed group and mixed-feeding group. *Bacteroidota* was at a low level for all the 3 groups in all periods. The ratio of *Firmicutes*/*Bacteroidota* was significantly higher in the formula-fed and mixed-fed groups than in the breast-fed group. The ratio of *Firmicutes*/*Bacteroidota* has been shown to be higher in the intestine of obese patients (36), and a previous study showed that breastfeeding can reduce the risk of overweight and obesity by 13% (37,38). The dominant genera in the formula-fed group in the second and third weeks were conditionally pathogenic bacteria, including *Streptococcus* and *Enterococcus*, and *Enterococcus* was also consistently the dominant genus in the mixed-feeding group in the first two weeks (39). In addition, a study showed that the relative abundance of *Enterococcus spp.* was high in the obese population (40). This suggests that mixed-feeding and formula-fed preterm infants may be at increased risk for the development of obesity later in life.
